# Comparison of three-dimensional tooth movements in virtual setups using different software packages for clear aligner therapy

**DOI:** 10.1186/s12903-025-05841-6

**Published:** 2025-04-11

**Authors:** Farah Y. Eid, Waddah Sabouni, Abbas Zaher, Mostafa A. Tageldin, Dina Elfouly

**Affiliations:** 1 Department of Orthodontics, Faculty of Dentistry, Alexandria University, Champolion street, Azarita, Alexandria Egypt; 2 Private practice, Bandol Rivage Orthodontics, Sanary-sur-Mer, France

**Keywords:** Software, Virtual setup, 3D tooth movement, Superimposition

## Abstract

**Background:**

With the advent of clear aligner therapy and their increasing popularity across the orthodontic field, many software packages have been introduced that implement the concept of virtual setups and consequently, the fabrication of in-house aligners. The objective of the present study was to evaluate and compare the differences in the resultant tooth movements when the same tooth movement values of virtual setups are implemented into four different software packages for clear aligner treatment, in patients with various degrees of dental crowding.

**Methods:**

Forty-four stereolithography (STL) files of adult Invisalign^®^-treated patients were included in this in silico study and were divided according to the amount of existent dental crowding into 2 groups; Moderate (2.5–5 mm), and Severe (> 5 mm). Initial STL files were imported into the other three tested software programs (Ortho Analyzer^®^, Maestro 3D Ortho Studio^®^^,^ Blue Sky Plan^®^), and the teeth were moved to replicate those performed in the virtual setup from ClinCheck^®^ Pro. The final outcome was then exported from the four software packages, with ClinCheck^®^ Pro STL files used as references, whereas those from the remaining software considered targets. Superimpositions were performed afterwards using Medit Link software between reference and target STL files to calculate the overall deviation.

**Results:**

Statistically significant deviation values were recorded between ClinCheck-Ortho Analyzer, and those recorded with both ClinCheck-Maestro and ClinCheck-Blue Sky in the moderate crowding group for the upper and lower models (*p* < 0.05). However, the differences in ClinCheck-Maestro and ClinCheck-Blue Sky deviation values were not statistically significant (*p* > 0.05). In the severe crowding group, statistically significant variances were documented among the investigated software programs, in contrast to the benchmark (*p* < 0.05). As per the reported outcomes, the highest deviation was attributed to ClinCheck-Ortho Analyzer, followed by ClinCheck-Blue Sky and ClinCheck-Maestro (*p* < 0.05).

**Conclusions:**

With the same tooth movement values provided, the four examined programs produced diverse final virtual tooth setups, with greater variance in cases of severe dental crowding. This deviation arises since every software operates using a distinct algorithm, with a different segmentation method and rotation center for the moving teeth.

## Background

Despite the increased popularity of clear aligner therapy over the past decade, the concept of tooth movement devoid of wires and attachments is not a novel one. It has been first described by Kesling in 1945 through his “Tooth Positioning Appliance”, where its use as a finishing device following fixed appliance therapy was its main purpose [1]. If used as advised, this device was believed to amend slight rotations, reduce existent spaces, and improve both arch form and teeth axial inclinations. Accordingly, a diagnostic wax setup was considered mandatory for proper appliance fabrication to help respect the biological limitations of tooth movements, to which clinicians should also be considerate of when constructing virtual setups in the current modern days [1].

Computer-aided design commenced in the 1950’s [2], and decades later, computer-aided design and manufacturing (CAD/CAM) systems were integrated into industrial planning and production, and eventually into the dental field through intra-oral scanners (IOSs) that capture digital optic impressions. It is noteworthy to mention that digital orthodontic models were found to have comparable accuracy to conventional plaster models, which in turn, advocates their use for clinical and research purposes [2, 3], together with the advantage of reducing patient discomfort.

In the 1990’s, Invisalign (Align Technology Inc., Santa Clara, CA) pioneered the use of CAD in orthodontic tooth movement through their custom-made clear aligners [4, 5]. Their primary focus was the treatment of mild crowding and spacing cases [6], but with the continuously evolving technology, it is now possible to correct more complex malocclusions [7, 8]. Consequently, Invisalign is now considered among the most recognized companies in clear aligner therapy in the orthodontic field, favored by many owing to its esthetic advantage.

In the recent years, various companies have implemented CAD/CAM technology, and adopted the concept of virtual tooth movement [9, 10]. This has been in the form of either a ready-made product such as clear aligners, or even through launching software platforms that allow practitioners to create their own virtual setups for the fabrication of in-house aligners or indirect bonding trays [11].

Each of the software packages available in the market implements different algorithm combinations and configurations of data input. Therefore, the aim of this research was to evaluate and compare the differences in the resultant tooth movements when similar tooth movement values are imported into four different software packages, including: ClinCheck^®^ Pro, Ortho Analyzer^®^, Maestro 3D Ortho Studio^®^, and Blue Sky Plan^®^, in subjects with various degrees of dental crowding. The null hypothesis was that there are no significant differences in the final tooth positions when the same tooth movement values are employed in all the tested software.

## Materials and methods

### Study subjects

An in silico study design was implemented for this investigation. Ethical approval was attained from the Institutional Review Board of the Faculty of Dentistry, Alexandria University, Alexandria, Egypt (IORG:0008839, Ethics Committee number 0823 − 12/2023). Adult patients who underwent Invisalign treatment were selected based on the following predetermined eligibility criteria: (1) Class I malocclusion. (2) Presence of a full permanent dentition, excluding third molars. (3) Availability of a ClinCheck from the SmartTrack era, where tooth movement tables are accessible. Exclusion criteria included: (1) History of facial trauma or craniofacial abnormalities. (2) Tooth impactions or malformations. (3) Discrepancy in centric occlusion/centric relation (CO/CR). The selected subjects were divided into two equal groups according to the severity of dental crowding, as follows: Moderate crowding (2.5–5 mm), and Severe crowding (> 5 mm) [12].

### Sample size calculation

The sample size was estimated to be assuming 80% power, and 5% alpha power. Eliliwi et al. [11] reported absolute average differences in tooth movements among different virtual setups. Based on comparison of means, the minimum sample size was calculated to be 20 patients and increased to 22 to make up for procedural problems. The total required sample size = number of groups × number per group = 2 × 22 = 44 patients [13]. Sample size calculation was performed using G*Power (Version 3.9.1.7).

### Experimental procedures

**Fig. 1:**
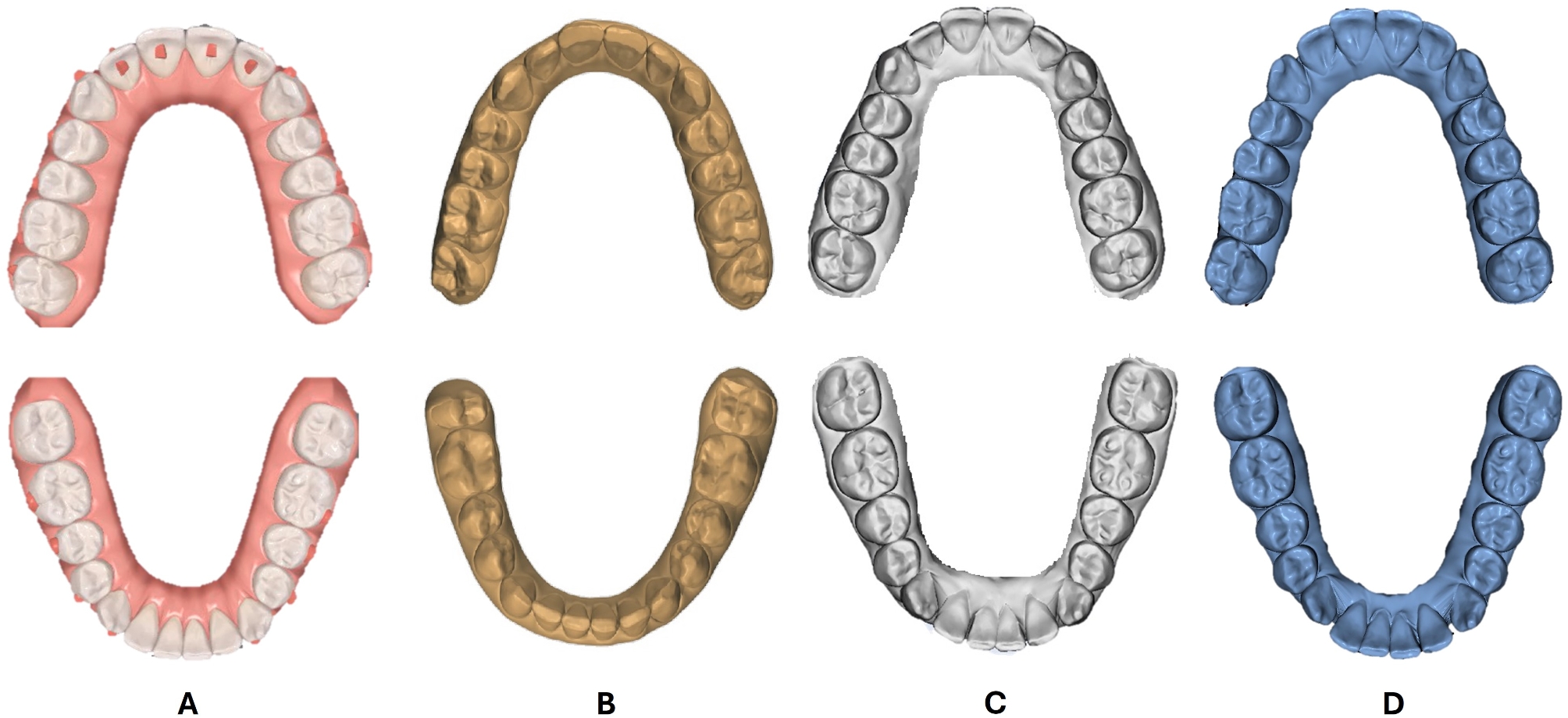
Upper and lower final virtual setups for moderate dental crowding cases in the tested software programs. **A**: ClinCheck^®^ Pro, **B**: Ortho Analyzer^®^, **C**: Maestro 3D Ortho Studio^®^, **D**: Blue Sky Plan^®^

**Fig. 2:**
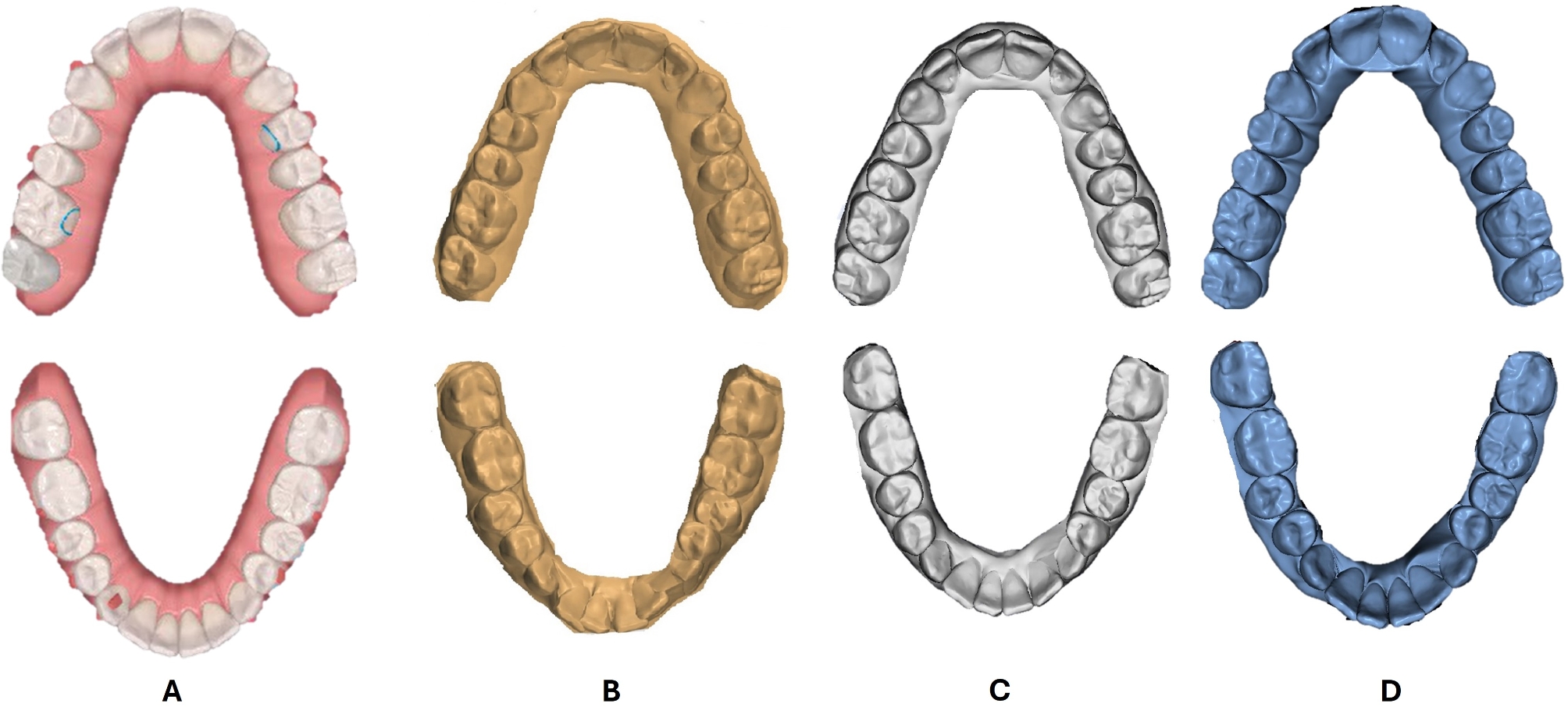
Upper and lower final virtual setups for severe dental crowding cases in the tested software programs. **A**: ClinCheck^®^ Pro, **B**: Ortho Analyzer^®^, **C**: Maestro 3D Ortho Studio^®^, **D**: Blue Sky Plan^®^

Following sample selection, the final stereolithography (STL) files of the cases decided upon from the approved ClinCheck were exported from ClinCheck^®^ Pro software (Align Technology Inc., Santa Clara, CA), together with the corresponding tooth movement tables, and interproximal reduction (IPR) values. The pre-treatment intra-oral scans for all the recruited subjects were performed by the same operator (**W.S.**). The initial pre-treatment STL files of all the study patients were procured and imported into the other three software packages: Ortho Analyzer^®^ (3Shape, Copenhagen, Denmark), Maestro 3D Ortho Studio^®^ (AGE Solutions^®^, Pontedera, Italy), and Blue Sky Plan^®^ (Blue Sky Bio, LLC, Illinois, US). In preparation for virtual digital setup, the imported pre-treatment maxillary and mandibular scans of the tested cases were oriented into three planes, followed by teeth segmentation. This involved the definition of the mesial and distal edges of the teeth and the creation of a cut spline for each tooth, that allows manual editing, if necessary. With the priorly determined occlusal and vertical planes contemplated as references, the long axes of the teeth were adjusted to ensure their ideal positioning. For each of the tested software, these preparatory steps were initially performed by one investigator, then checked and agreed upon by another investigator before proceeding to the step of virtual tooth movement (**D.E.**, **F.E.**, **M.T.**), dictated by each patient’s ClinCheck tooth movement table. The implemented movements were both linear and angular, such as Extrusion/Intrusion (mm), Translation Buccal/Lingual (mm), Translation Mesial/Distal (mm), Rotation (º), Angulation (º), and Inclination (º). The values from the ClinCheck tables, along with the relevant IPR values were accurately duplicated in each of the three software packages in the two study groups (Figs. 1, and 2).

### Outcomes’ measurement

**Fig. 3:**
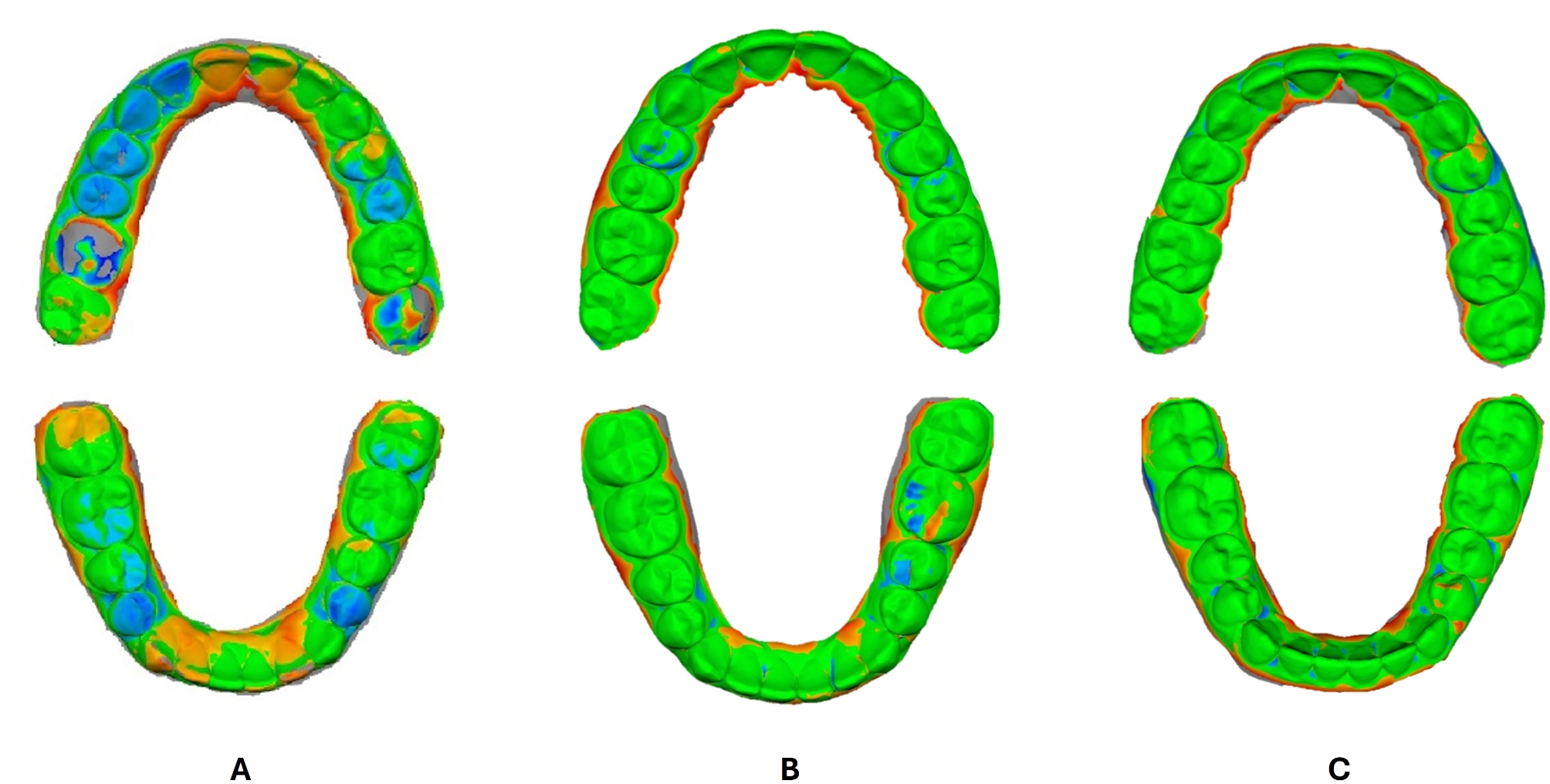
Color-difference maps of upper and lower models in moderate crowding cases. **A**: ClinCheck-Ortho Analyzer, **B**: ClinCheck-Maestro, **C**: ClinCheck-Blue Sky

**Fig. 4:**
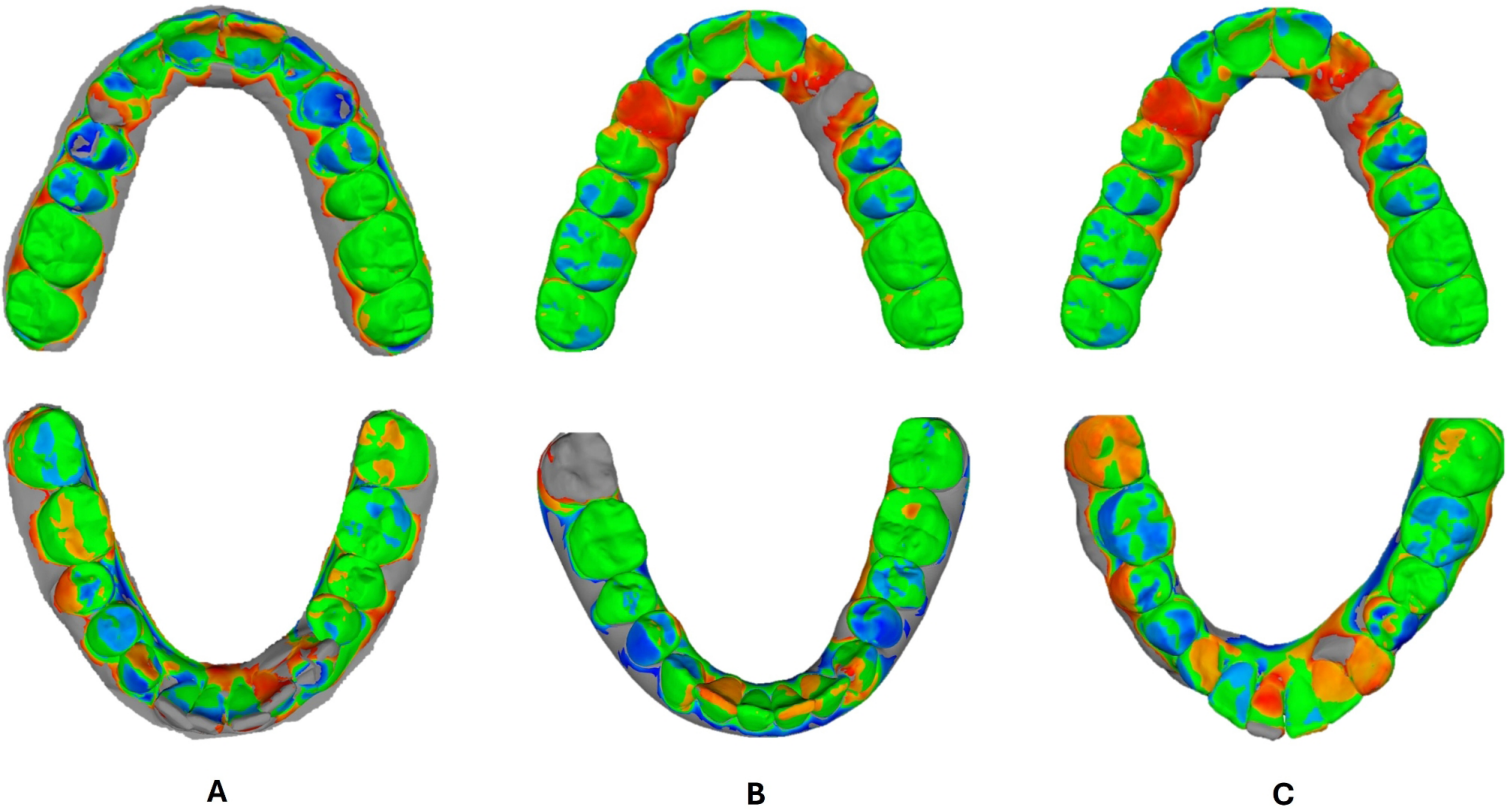
Color-difference maps of upper and lower models in severe crowding cases. **A**: ClinCheck-Ortho Analyzer, **B**: ClinCheck-Maestro, **C**: ClinCheck-Blue Sky

Final outcomes of both maxillary and mandibular virtual model setups from the four software packages were exported and saved as STL files. For the deviation analysis using the non-metrology grade freeware program Medit Design App., a component of Medit Link software (MEDIT Corp., Seoul, South Korea), superimpositions were performed for both the moderate and the severe crowding groups **(**Figs. 3, and 4**)**. ClinCheck^®^ Pro final STL files were regarded as references, whereas those from the remaining three software were considered targets. The “Automatic alignment tool” of the freeware software was employed for the automatic alignment of the reference and target data. Color-difference maps were then generated using the deviation display mode of the freeware program, where the minimum and maximum deviation values were set to -1 mm and + 1 mm, with a tolerance range of -0.025 mm and + 0.025 mm. The resultant estimates from the color-difference maps reflected how far the calculated deviations were from zero between the reference and the target, hence they allowed the evaluation of overall differences between specific points. The recorded differences in terms of Absolute Average (Abs Avg.), Positive Average (+ Avg.), and Negative Average (− Avg.) values for the upper and lower models were automatically calculated and compared between the superimpositions of ClinCheck-Ortho Analyzer, ClinCheck-Maestro, and ClinCheck-Blue Sky for cases of both moderate and severe dental crowding.

### Blinding

Due to the nature of the study, the operator could not have been blinded during the assessment of each of the tested software packages. However, the statistician was blinded throughout the data assessment process.

### Reliability

Intra-examiner reliability for the recorded deviation values was assessed by randomly selecting 10 STL patient files from each of the study groups and repeating the superimposition steps after two weeks by the same researcher on Medit Link software (**D.E.**, **M.T.**). Calculation of intra-class correlation coefficients (ICC) was performed.

### Statistical analysis

Descriptive statistics were calculated as means, standard deviation (SD), medians, interquartile range (IQR), and 95% confidence intervals (CI)s. Normality was tested using descriptive statistics, histograms, Q-Q plots, and Kolmogorov–Smirnov normality test. All data showed normal distribution, so One-way ANOVA was used for comparisons between the study groups, followed by multiple pairwise comparisons using Bonferroni adjusted significance. Data were analyzed using IBM SPSS for Windows (V.26), and significance level was set at p-value < 0.05.

## Results

**Fig. 5:**
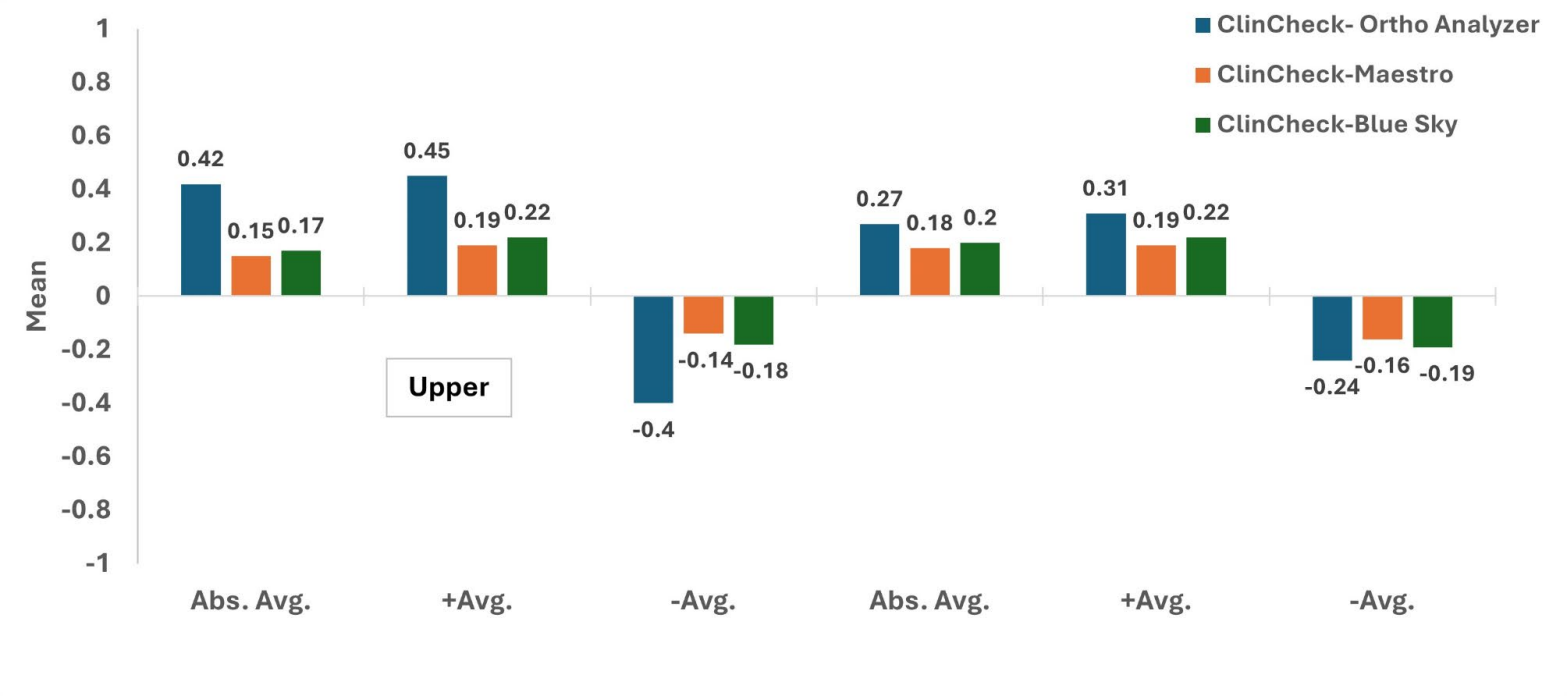
Comparison between the recorded deviation values in the three tested software with ClinCheck^®^ Pro as the reference in moderate crowding cases

Intra-examiner reliability ICC scores for the measured outcomes ranged between 0.86 and 0.98 which was considered excellent [14]. Tables 1 and 2 represent the documented deviation values in the moderate crowding group, where differences among Abs Avg., + Avg., and − Avg. for the upper and lower models revealed significantly higher values upon comparing the recorded deviation numbers of ClinCheck-Ortho Analyzer, with those of both ClinCheck-Maestro and ClinCheck-Blue Sky (*p* < 0.05) **(**Fig. 5**)**. However, the differences between ClinCheck-Maestro and ClinCheck-Blue Sky deviation values were non-significant with regards to the same variables (*p* > 0.05), in both the upper and lower model scans.

**Table 1:** Comparison between the three software programs in the moderate crowding group

			ClinCheck- Ortho Analyzer	ClinCheck-Maestro	ClinCheck-Blue Sky	*P* value
**Upper**	**Abs. Avg.**	**Mean (SD)**	0.42 (0.12) **a**	0.15 (0.04) **b**	0.17 (0.03) **b**	< 0.001*
		**Median (IQR)**	0.40 (0.30, 0.53)	0.15 (0.12, 0.17)	0.18 (0.15, 0.19)	
		**Min– Max**	0.25–0.65	0.09–0.22	0.10–0.20	
		**95% CI**	0.36, 0.46	0.13, 0.16	0.15, 0.18	
	**+Avg.**	**Mean (SD)**	0.45 (0.13) **a**	0.19 (0.05) **b**	0.22 (0.03) **b**	< 0.001*
		**Median (IQR)**	0.42 (0.33, 0.55)	0.17 (0.14, 0.20)	0.22 (0.21, 0.23)	
		**Min– Max**	0.27–0.69	0.11–0.30	0.11–0.29	
		**95% CI**	0.38, 0.50	0.16, 0.20	0.21, 0.24	
	**-Avg.**	**Mean (SD)**	-0.40 (0.12) **a**	-0.14 (0.05) **b**	-0.18 (0.06) **b**	< 0.001*
		**Median (IQR)**	-0.39 (-0.49, -0.30)	-0.14 (-0.16, -0.11)	-0.19 (-0.22, -0.15)	
		**Min– Max**	-0.67– -0.22	-0.29– -0.06	-0.28– -0.07	
		**95% CI**	-0.46, -0.35	-0.16, -0.12	-0.21, -0.16	
**Lower**	**Abs. Avg.**	**Mean (SD)**	0.27 (0.05) **a**	0.18 (0.03) **b**	0.20 (0.05) **b**	< 0.001*
		**Median (IQR)**	0.28 (0.23, 0.31)	0.19 (0.16, 0.20)	0.19 (0.17, 0.23)	
		**Min– Max**	0.18–0.35	0.12–0.24	0.09–0.30	
		**95% CI**	0.25, 0.29	0.16, 0.19	0.17, 0.22	
	**+Avg.**	**Mean (SD)**	0.31 (0.07) **a**	0.19 (0.04) **b**	0.22 (0.05) **b**	< 0.001*
		**Median (IQR)**	0.32 (0.27, 0.35)	0.20 (0.17, 0.22)	0.23 (0.19, 0.25)	
		**Min– Max**	0.18–0.48	0.11–0.26	0.11–0.36	
		**95% CI**	0.27, 0.33	0.17, 0.21	0.20, 0.25	
	**-Avg.**	**Mean (SD)**	-0.24 (0.08) **a**	-0.16 (0.04) **b**	-0.19 (0.07) **b**	0.001*
		**Median (IQR)**	-0.26 (-0.30, -0.20)	-0.17 (-0.20, -0.13)	-0.18 (-0.23, -0.14)	
		**Min– Max**	-0.36– -0.11	-0.25– -0.10	-0.31– -0.07	
		**95% CI**	-0.28, -0.21	-0.19, -0.15	-0.22, -0.16	

SD: Standard Deviation, IQR: Interquartile Range, Min: Minimum, Max: Maximum, CI: Confidence Interval

One-way ANOVA was used

*statistically significant at p-value < 0.05

a-c: different letters denote significant differences between groups using Bonferroni correction

**Table 2:** Post-hoc comparisons between the three software programs in the moderate crowding group

		Group	Compared to	*P* value
**Upper**	**Abs. Avg.**	**ClinCheck- Ortho Analyzer**	**ClinCheck-Maestro**	< 0.001*
			**ClinCheck-Blue Sky**	< 0.001*
		**ClinCheck-Maestro**	**ClinCheck-Blue Sky**	1.00
	**+Avg.**	**ClinCheck- Ortho Analyzer**	**ClinCheck-Maestro**	< 0.001*
			**ClinCheck-Blue Sky**	< 0.001*
		**ClinCheck-Maestro**	**ClinCheck-Blue Sky**	0.40
	**-Avg.**	**ClinCheck- Ortho Analyzer**	**ClinCheck-Maestro**	< 0.001*
			**ClinCheck-Blue Sky**	< 0.001*
		**ClinCheck-Maestro**	**ClinCheck-Blue Sky**	0.31
**Lower**	**Abs. Avg.**	**ClinCheck- Ortho Analyzer**	**ClinCheck-Maestro**	< 0.001*
			**ClinCheck-Blue Sky**	0.003*
		**ClinCheck-Maestro**	**ClinCheck-Blue Sky**	0.59
	**+Avg.**	**ClinCheck- Ortho Analyzer**	**ClinCheck-Maestro**	< 0.001*
			**ClinCheck-Blue Sky**	< 0.001*
		**ClinCheck-Maestro**	**ClinCheck-Blue Sky**	0.19
	**-Avg.**	**ClinCheck- Ortho Analyzer**	**ClinCheck-Maestro**	< 0.001*
			**ClinCheck-Blue Sky**	0.03*
		**ClinCheck-Maestro**	**ClinCheck-Blue Sky**	0.56

*statistically significant using Bonferroni correction

**Fig. 6:**
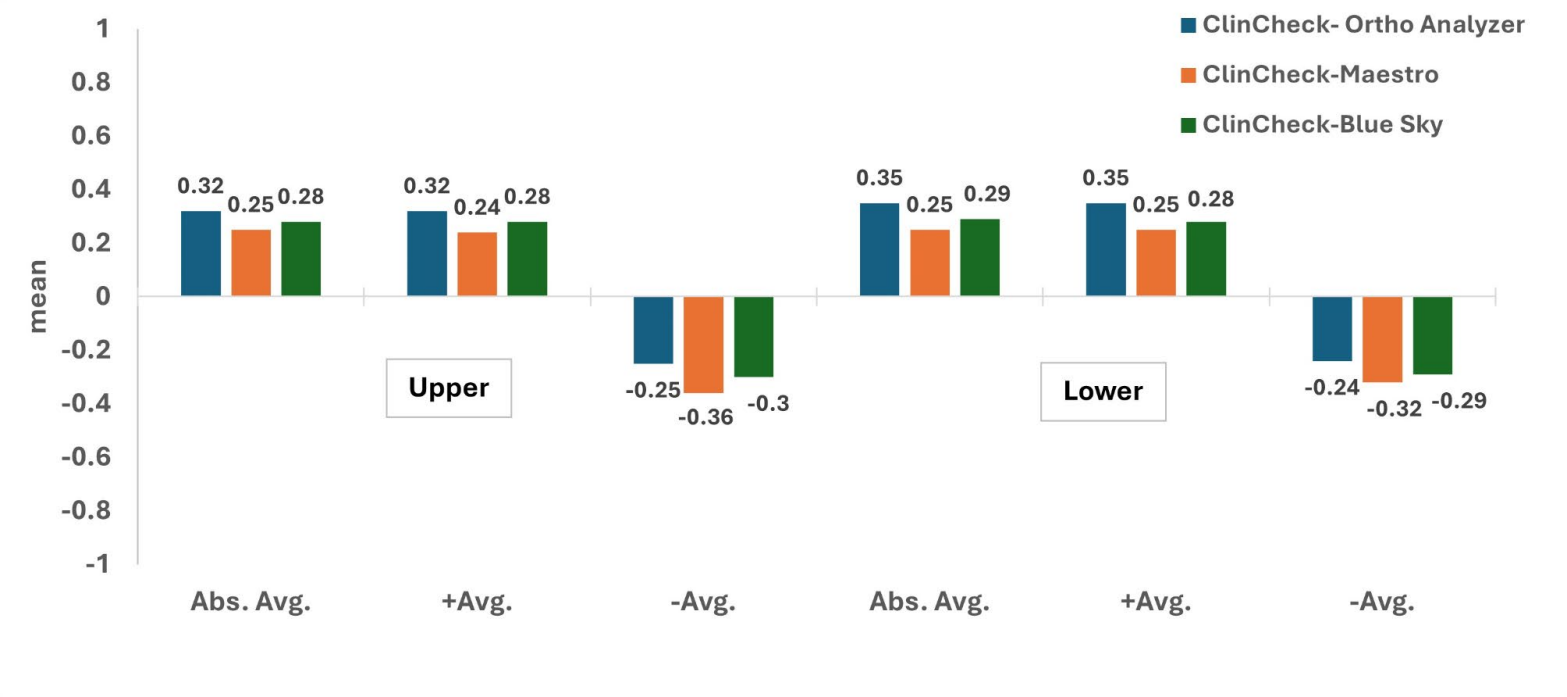
Comparison between the recorded deviation values in the three tested software with ClinCheck^®^ Pro as the reference in severe crowding cases

In Tables 3 and 4, the recorded Abs Avg., + Avg., and − Avg. values for both upper and lower scans in cases with severe dental crowding are depicted, with statistically significant differences being reported among the investigated software programs regarding the recorded deviations; ClinCheck-Ortho Analyzer, ClinCheck-Maestro, and ClinCheck-Blue Sky (*p* < 0.05) **(**Fig. 6**)**. Results also showed that ClinCheck-Ortho Analyzer had the highest values, followed by ClinCheck-Blue Sky, and finally ClinCheck-Maestro (*p* < 0.05).

**Table 3:** Comparison between the three software programs in the severe crowding group

			ClinCheck- Ortho Analyzer	ClinCheck-Maestro	ClinCheck-Blue Sky	*P* value
**Upper**	**Abs. Avg.**	**Mean (SD)**	0.32 (0.06) **a**	0.25 (0.03) **b**	0.28 (0.03) **c**	< 0.001*
		**Median (IQR)**	0.35 (0.28, 0.38)	0.25 (0.22, 0.28)	0.28 (0.26, 0.31)	
		**Min– Max**	0.21–0.39	0.20–0.29	0.22–0.34	
		**95% CI**	0.30, 0.35	0.23, 0.26	0.27, 0.30	
	**+Avg.**	**Mean (SD)**	0.32 (0.07) **a**	0.24 (0.03) **b**	0.28 (0.03) **c**	< 0.001*
		**Median (IQR)**	0.34 (0.24, 0.38)	0.24 (0.23, 0.25)	0.28 (0.26, 0.30)	
		**Min– Max**	0.21–0.40	0.20–0.29	0.21–0.33	
		**95% CI**	0.28, 0.35	0.23, 0.25	0.27, 0.29	
	**-Avg.**	**Mean (SD)**	-0.25 (0.03) **a**	-0.36 (0.03) **b**	-0.30 (0.03) **c**	< 0.001*
		**Median (IQR)**	-0.26 (-0.28, -0.24)	-0.37 (-0.38, -0.35)	-0.31 (-0.32, -0.27)	
		**Min– Max**	-0.29– -0.20	-0.39– -0.30	-0.35– -0.24	
		**95% CI**	-0.27, -0.24	-0.37, -0.35	-0.31, -0.28	
**Lower**	**Abs. Avg.**	**Mean (SD)**	0.35 (0.04) **a**	0.25 (0.03) **b**	0.29 (0.02) **c**	< 0.001*
		**Median (IQR)**	0.35 (0.32, 0.38)	0.26 (0.22, 0.28)	0.29 (0.27, 0.30)	
		**Min– Max**	0.27–0.39	0.20–0.30	0.26–0.32	
		**95% CI**	0.34, 0.37	0.24, 0.27	0.28, 0.30	
	**+Avg.**	**Mean (SD)**	0.35 (0.03) **a**	0.25 (0.03) **b**	0.28 (0.04) **c**	< 0.001*
		**Median (IQR)**	0.34 (0.32, 0.38)	0.24 (0.22, 0.29)	0.28 (0.35, 0.32)	
		**Min– Max**	0.30–0.340	0.21–0.32	0.22–0.34	
		**95% CI**	0.33, 0.36	0.24, 0.27	0.27, 0.30	
	**-Avg.**	**Mean (SD)**	-0.24 (0.03) **a**	-0.32 (0.04) **b**	-0.29 (0.04) **c**	< 0.001*
		**Median (IQR)**	-0.24 (-0.26, -0.21)	-0.32 (-0.35, -0.31)	-0.29 (-0.32, -0.25)	
		**Min– Max**	-0.30– -0.20	-0.39– -0.22	-0.33– -0.23	
		**95% CI**	-0.25, -0.22	-0.34, -0.30	-0.30, -0.27	

SD: Standard Deviation, IQR: Interquartile Range, Min: Minimum, Max: Maximum, CI: Confidence Interval

One-way ANOVA was used

*statistically significant at p-value < 0.05

a-b: different letters denote significant differences between groups using Bonferroni correction

**Table 4:** Post-hoc comparisons between the three software programs in the severe crowding group

		Group	Compared to	*P* value
**Upper**	**Abs. Avg.**	**ClinCheck- Ortho Analyzer**	**ClinCheck-Maestro**	< 0.001*
			**ClinCheck-Blue Sky**	0.007*
		**ClinCheck-Maestro**	**ClinCheck-Blue Sky**	0.04*
	**+Avg.**	**ClinCheck- Ortho Analyzer**	**ClinCheck-Maestro**	< 0.001*
			**ClinCheck-Blue Sky**	0.04*
		**ClinCheck-Maestro**	**ClinCheck-Blue Sky**	0.03*
	**-Avg.**	**ClinCheck- Ortho Analyzer**	**ClinCheck-Maestro**	< 0.001*
			**ClinCheck-Blue Sky**	< 0.001*
		**ClinCheck-Maestro**	**ClinCheck-Blue Sky**	< 0.001*
**Lower**	**Abs. Avg.**	**ClinCheck- Ortho Analyzer**	**ClinCheck-Maestro**	< 0.001*
			**ClinCheck-Blue Sky**	< 0.001*
		**ClinCheck-Maestro**	**ClinCheck-Blue Sky**	0.001*
	**+Avg.**	**ClinCheck- Ortho Analyzer**	**ClinCheck-Maestro**	< 0.001*
			**ClinCheck-Blue Sky**	< 0.001*
		**ClinCheck-Maestro**	**ClinCheck-Blue Sky**	0.03*
	**-Avg.**	**ClinCheck- Ortho Analyzer**	**ClinCheck-Maestro**	< 0.001*
			**Clincheck-Blue Sky**	< 0.001*
		**ClinCheck-Maestro**	**Clincheck-Blue Sky**	0.004*

*statistically significant using Bonferroni correction

## Discussion

Pertaining to the rise of clear aligner therapy in orthodontics, and the consequent emergence of various software programs addressing the concept of virtual tooth movement, the purpose of this investigation was to compare the final outcomes of four distinct virtual setup software packages after implementing the exact amounts of tooth movements in all directions, in subjects with different degrees of dental crowding. The tested software were Ortho Analyzer^®^, Maestro 3D Ortho Studio^®^, and Blue Sky Plan^®^, in comparison with ClinCheck^®^ Pro as the benchmark. As per the reported outcomes, statistically significant differences have been recorded between the tested software, hence the null hypothesis was rejected.

ClinCheck^®^ Pro by Invisalign was considered the reference in the present study since it is considered one of the recognized companies in clear aligner therapy in the orthodontic field, with the remaining tested software commonly employed in planning in-office aligners and are usually compared to that by Invisalign. Moreover, similar studies comparing software packages for in-office aligners’ fabrication have also used ClinCheck^®^ Pro as a reference for comparison, including that by Eliliwi et al. [15].

For a precise tooth movement appraisal among the evaluated software, digital superimpositions are mandatory, as currently considered in contemporary orthodontics. A quantified superimposition enables practitioners to acknowledge the limitations as well as the capabilities of the employed mechanics [16–18]. Software packages vary in their registration methods, in their three-dimensional (3D) tooth movement assessment methods, in addition to the cost, time and intricacy required to undergo certain procedures [19, 20]. Metrology-grade software have been the tools of choice in dental research for the 3D analysis of digital impressions and restorations. However, expertise and high cost are both indispensable with the operation of these software, since they commonly require a specialized training course pertaining to their complexity. In consequence, a non-metrology grade 3D analysis software has been introduced (Medit Link; MEDIT Corp., Seoul, South Korea) with the advantage of it being a freeware program that does not entail financial burdens, in addition to being user-friendly [21, 22]. More importantly, equivalent performances have been reported using both metrology-grade and non-metrology grade software when recording deviation values [21], and specifically in overall deviation measurements [23], hence its employment in the present study.

Several methods for target and reference alignment could be employed using Medit Link software, such as “Automatic alignment”, “Manual alignment”, and “Alignment with selected area” [24]. The chosen method in the present study was the “Automatic alignment” which is an artificial intelligence-based (AI) method that calculates the best possible matching points between the tested surfaces, devoid of human intervention. Since, it has been reported that the “Automatic alignment” method showed deviation values in accordance with the other alignment methods with non-significant differences [24], hence, it all goes back to convenience and operator preference.

Despite the fact that the teeth were virtually moved in Ortho Analyzer^®^, Maestro 3D Ortho Studio^®^, and Blue Sky Plan^®^ replicating the numbers imported from the ClinCheck^®^ Pro tooth movement tables, the final outcomes were significantly different in the other software programs although one could expect the opposite. The aftermath of this observation will be reflected when operators switch to in-house aligners, where difficulties may be encountered when they tend to use different software programs and compare the resultant values.

As per the reported results in the moderate crowding group, the greatest deviation has been attributed to ClinCheck-Ortho Analyzer, in contrast to the other two tested software with the designated reference. A possible cause to this observation could be the difference in the centers of rotation between the investigated software packages when altering teeth inclinations. ClinCheck^®^ Pro utilizes a point 1–2 mm from the cemento-enamel junction (CEJ) as the center of rotation [11], whereas Maestro 3D Ortho Studio^®^’s default setting places it at a point approximately in the center of tooth clinical crown. Both Ortho Analyzer^®^ [11] and Blue Sky Plan^®^ consider the center of rotation as a point at the apex of tooth root.

Despite the statistical non-significance of the recorded deviation between Maestro 3D Ortho Studio^®^ and Blue Sky Plan^®^ in contrast to the decided reference, the deviation values documented with Maestro 3D Ortho Studio^®^ were the least, and therefore, its outcomes were considered the closest to those of ClinCheck^®^ Pro, an observation that could be appertained to the fact that the center of rotation utilized for tooth movement by Maestro 3D Ortho Studio^®^ is close to that contemplated by ClinCheck^®^ Pro, both of which are related to the crowns of the teeth, that are in fact, actual structures recorded in the original pre-treatment scan.

Ortho Analyzer^®^ and Blue Sky Plan^®^ both contemplated the root apices as the rotation centers of the moving teeth as previously stated, nevertheless, the greatest deviation has been depicted with Ortho Analyzer^®^ in contrast to ClinCheck^®^ Pro, whereas non-significant differences were found in the ClinCheck-Blue Sky deviation values. This distinct observation owes back to the ability of each software to estimate the root lengths of the teeth, which could vary greatly in their morphology, especially their lengths, and in turn affect the estimated tooth movement. The longer the imaginary root automatically predicted by the software, the farther away it is from the rotation center considered by ClinCheck^®^ Pro, and therefore, the greater the deviation between the reference and the target in the resultant tooth movement. Recent software developments therefore make it possible to incorporate cone beam computed tomography (CBCT) images, and superimpose them on the STL files of patients’ scans in an effort to overcome this downside and to allow the precise definition of the teeth centroids, which in turn, improves the accuracy of the predicted root positions as well as the anticipated tooth movements [11].

Corroborating our results, were those reported by Eliliwi et al. [15], where the greatest difference in their software comparisons, was that between ClinCheck^®^ Pro and Ortho Analyzer^®^. To the best of our knowledge, no studies have tested the accuracy of tooth movements performed using Maestro 3D Ortho Studio^®^ and Blue Sky Plan^®^, with reference to ClinCheck^®^ Pro, thus, the outcomes of these software could not be compared to others.

Regarding cases with severe dental crowding, a difference of statistical significance was attained between the three software in question, in comparison to the reference: ClinCheck^®^ Pro. A plausible explanation could go back to the dissimilarity in the employed segmentation methods, where ClinCheck^®^ Pro undergoes an automatic AI-based segmentation process [15], unlike the remainder of the tested software where manual segmentation and preparation of the teeth were performed by the operator prior to the final virtual tooth movement step. In a study by Zhang et al. [25], automatic segmentation was reported to be of inferior quality, especially in cases of tooth malformations, since the process is mainly dependent on the geometric characteristics of the scanned teeth. Moreover, He et al. [26] reported a lower clinical performance by a machine learning segmentation method because of the discrepancy in the morphology of the teeth. Given the obvious teeth rotations and the limited access to the exact borders of the teeth in specific areas in most of the severe crowding cases posing a challenge to operators with manual segmentation, in addition to the questionable accuracy of automated segmentation that relies mainly on the morphology of the teeth, the significant differences between the tested software and ClinCheck^®^ Pro could be explicated owing to the possibility of faulty AI-driven segmentation in severe dental crowding.

Another prime factor contributing to the reported results in the current study in both the moderate and the severe crowding cases may be the difference in the algorithms operating each of the investigated software, since the exact modi operandi of the software have not been disclosed by the providers, which could also influence the generalizability of the study outcomes.

### Study limitations

In consequence to the absence of the teeth roots in the acquired intra-oral scans, the centroid of each moved tooth could not be determined, and therefore, the implemented movements were based solely on the anatomy of the clinical crowns, which could pose significant errors in cases of disharmony between the long axis of the root and that of the crown of each moving unit.

### Recommendations

The reported significant software differences in cases of severe dental crowding, could possibly affect the predictability of the treatment outcome. Therefore, a second scan with a second segmentation process is recommended after a few months of treatment and relative alignment of the teeth, to overcome this downside and to enhance the outcome of orthodontic treatment.

## Conclusions

With the same tooth movement values provided, the four examined programs produced diverse final virtual tooth setups, with greater variance in cases of severe dental crowding. This deviation arises since every software operates using a distinct algorithm, with a different segmentation method and rotation centers for the moving teeth. Therefore, with the increasing popularity of in-house aligners taken into account, in addition to the subsequent upsurge in software packages that offer virtual setup options, the reported outcomes should be considered by clinicians upon choosing the appropriate software.

## Data Availability

The datasets used and/or analyzed during the current study are available from the corresponding author on reasonable request.

## Abbreviations

CAD/CAM: Computer-aided design and manufacturing
CO/CR: Centric occlusion/centric relation
STL: Stereolithography
IPR: Interproximal reduction
Abs Avg.: Absolute Average
+Avg.: Positive Average
-Avg.: Negative Average
ICC: Intra-class correlation coefficient
SD: Standard deviation
IQR: Interquartile range
CI: Confidence intervals
AI: Artificial intelligence
CEJ: Cemento-enamel junction
CBCT: Cone-beam computed tomography
3D: Three-dimensional
